# Potentially Toxic Elements in Phosphate Processing: A Comparative Assessment of Solid Wastes and Effluents from Beneficiation and Fertilizer Production in Southwest Tunisia

**DOI:** 10.3390/toxics14050438

**Published:** 2026-05-15

**Authors:** Elhem Bouchiba, Ariadna Verdaguer, Cristian-Gómez Canela, Eduardo Alberto López-Maldonado, Mohamed Ali Borgi

**Affiliations:** 1 Laboratory of Biotechnology and Bio-Monitoring of the Environment and Oasis Ecosystems (LBBEEO), Faculty of Sciences of Gafsa, University of Gafsa, Zarroug, Gafsa 2112, Tunisia; ebouchiba482@gmail.com; 2 Department of Analytical and Applied Chemistry, School of Engineering, Institut Químic of Sarrià-University of Ramon Llull, Via Augusta 390, 08017 Barcelona, Spain; ariadna.verdaguer@iqs.url.edu (A.V.);; 3 Faculty of Chemical Sciences and Engineering, Autonomous University of Baja California, Tijuana 22424, Mexico

**Keywords:** industrial effluents, potentially toxic elements (PTEs), phosphate-processing wastes, ecological risk assessment, human health risk assessment, phosphogypsum

## Abstract

This study investigates the environmental and health impacts of potentially toxic elements (PTEs) in solid and liquid wastes from phosphate beneficiation and fertilizer production in the Mdhilla area, Gafsa Basin, Tunisia. Solid wastes, including phosphate tailings (PTs) and phosphogypsum (PG), and associated industrial effluents from phosphate beneficiation (PBE) and fertilizer production (PFE), were characterized using physicochemical analysis, ICP-MS, SEM–EDX, and ion chromatography. Single and integrated pollution indices, along with conservative human health risk assessments, were applied to evaluate cumulative contamination and potential risks. PT exhibited near-neutral pH (7.64) and high PTE enrichment (Zn 350 mg kg^−1^, Cr 329 mg kg^−1^, Cd 38.8 mg kg^−1^), whereas PG was strongly acidic (pH 3.13) and comparatively depleted in metals, reflecting process-dependent partitioning. Despite neutral pH, PBEs contained markedly higher metal concentrations than PFEs, with Fe (163 mg L^−1^), Cr (3.09 mg L^−1^), Cd (0.49 mg L^−1^), and Pb (0.71 mg L^−1^) exceeding discharge limits. Pollution indices indicated severe to extreme contamination, with PBE showing an exceptionally high contamination degree (C_deg_ = 63,659) compared to PFE (C_deg_ = 12,815), and elevated Toxic Element Pollution Index (PTEPI) values confirmed stronger cumulative pollution in PBE. Potential ecological risk indices (PERI > 600) revealed very high ecological risk for both effluents, primarily driven by Cd, Co, and Tl. Although dermal contact may represent the most frequent exposure route, risk assessment results indicated that accidental oral ingestion is the dominant pathway contributing to both non-carcinogenic and carcinogenic risks, with children being particularly vulnerable. Non-carcinogenic risk thresholds (HQ > 1) were exceeded for PBE, while total carcinogenic risks approached or exceeded regulatory limits (10^−6^–10^−4^), mainly due to Cd and Cr. Overall, phosphate beneficiation was the primary source of cumulative metal loading and associated ecological and health risks, while fertilizer production partially reduced contamination. These findings underscore the urgent need for improved management and mitigation of phosphate-processing wastes in industrial regions.

## 1. Introduction

The environmental landscape has undergone significant alterations in recent decades due to the rapid intensification of anthropogenic activities worldwide [1]. Industrial development, urban expansion, and agricultural practices generate large volumes of waste byproducts, including industrial effluents, domestic sewage, and agricultural runoff. Many of these byproducts contain hazardous substances that ultimately contaminate natural ecosystems, particularly the hydrosphere. In developing countries, rapid industrialization, combined with insufficient regulatory enforcement, has accelerated the deterioration of water quality, with effluent discharge standards frequently being violated or inadequately monitored [2,3]. As a result, untreated or poorly treated industrial wastewater is often released directly into the receiving environments. These effluents are widely recognized as complex and highly toxic mixtures of pollutants because they contain diverse chemical contaminants released at multiple stages of industrial processes [4]. Of particular concern are potentially toxic elements (PTEs), which are persistent, non-biodegradable, highly toxic, and prone to bioaccumulation and biomagnification along food chains [5]. The presence of PTEs in aquatic systems can severely disrupt ecosystem functioning and pose serious risks to human health [6]. Phosphate mining, beneficiation, and fertilizer manufacturing are among the industrial sectors most frequently implicated in the contamination of water resources by PTEs [7,8]. Phosphate rock is globally recognized as a natural repository of PTEs, including Cd, Pb, Cr, Zn, Cu, and As [9]. During phosphate extraction and processing, these elements are redistributed among solid waste and liquid effluents. Environmental contamination related to phosphate industries has been widely reported in major phosphate-producing countries such as Tunisia, Morocco, China, Jordan, Egypt, and the United States. There, elevated metal concentrations in effluents, soils, and sediments have been associated with increased ecological and human health risks [6,10,11,12,13]. However, most existing studies have focused on phosphate mining and beneficiation residues or on fertilizer industry effluents independently, while integrated assessments comparing both types of effluent remain scarce, particularly in North African phosphate basins. Tunisia is one of the world’s leading phosphate producers, where the Gafsa Phosphate Company (GPC) and the Tunisian Chemical Group (TCG) jointly manage phosphate extraction and transformation. Unlike most phosphate-producing regions, which specialize in either mining or fertilizer production, the Mdhilla area (Gafsa Basin) uniquely combines phosphate beneficiation and fertilizer manufacturing within a confined geographical zone. Phosphate rock undergoes extensive beneficiation processes, including crushing, grinding, scrubbing, flotation, and gravity or magnetic separation, to increase its P_2_O_5_ content before chemical processing [14]. The upgraded phosphate is then treated using the wet process with sulfuric acid to produce phosphoric acid or triple superphosphate (TSP). This stage is known to further mobilize PTEs into solid and liquid byproducts [13]. Both beneficiation and fertilizer production generate large quantities of solid wastes, including phosphate tailings (PTs) and phosphogypsum (PG). These materials are the primary sources of PTEs within the industrial system [9]. These solid matrices are fundamental sources of metals released into associated liquid effluents through mechanical disturbance, particle entrainment, surface leaching, and chemical dissolution during processing. Thus, the physicochemical characteristics and metal content of PT and PG directly influence the composition of phosphate beneficiation effluents (PBEs) and phosphate fertilizer effluents (PFEs). Therefore, characterizing solid waste is essential for understanding the partitioning of metals between the solid and liquid phases, as well as for elucidating the origin and behavior of contaminants observed in industrial wastewaters. Previous studies conducted in the Gafsa phosphate basin have reported elevated concentrations of PTEs in beneficiation wastewaters discharged from several phosphate beneficiation plants, including Moulares, Redeyef, Metloui, and Mdhilla [8,15,16,17,18]. However, data on the metal composition of effluents released from phosphate fertilizer plants remain limited. In a previous study, we reported that untreated effluents from the fertilizer production industry contain significant levels of toxic elements and exhibit acute biological toxicity, raising concerns regarding environmental and human exposure [19]. Due to the proximity of both industrial units to residential areas and the continuous discharge of untreated effluents over long surface pathways, local populations, including children, workers, and nearby residents, are at a heightened risk of repeated contact with contaminated waters. Assessing PTE concentrations alone is insufficient to fully characterize environmental pollution or associated health risks fully. Accordingly, applying pollution indices and risk assessment models, such as the contamination factor (CF), pollution index (PI), degree of contamination (C_deg_), potential ecological risk index (PERI), and human health risk assessment (HRA), provides a robust and integrative framework for evaluating both single and multi-element contamination scenarios [12,20]. These indices enable the discrimination between geochemical enrichment, regulatory exceedance, cumulative pollution pressure, and potential ecological and human health impacts. Despite the numerous studies that have reported PTE contamination in phosphate mining areas of southern Tunisia and other phosphate-producing regions, the majority of investigations have addressed solid waste, soil, sediment, and industrial effluents as discrete environmental compartments. Specifically, phosphate beneficiation residues and fertilizer industry effluents have generally been examined independently, without an explicit linkage of solid waste characteristics to effluent composition, cumulative pollution indices, ecological risk, and human health implications within a unified framework. Furthermore, comparative assessments distinguishing contamination and risk profiles between phosphate beneficiation and fertilizer production effluents within the same industrial setting are scarce. The present study addresses a critical gap in the literature by jointly characterizing solid waste (phosphate tailings and phosphogypsum) and its associated industrial effluents. This study integrates physicochemical analysis with single and cumulative pollution indices, ecological risk assessment, and human health risk evaluation. This provides a more comprehensive understanding of contamination dynamics in phosphate-processing environments. The objectives of this study are: (i) to characterize the physicochemical properties and PTE content of solid wastes (PT and PG) and associated industrial effluents (PBE and PFE); (ii) to evaluate contamination levels using single and integrated pollution indices; (iii) to assess potential ecological risks; and (iv) to estimate non-carcinogenic and carcinogenic human health risks associated with dermal and oral exposure pathways. This integrated approach aims to provide a scientific basis for environmental management strategies and risk mitigation in phosphate-processing regions. Unlike the majority of previous studies that focused on either solid wastes or liquid effluents independently, the present work offers a unified assessment of potentially toxic elements across both solid and liquid phases within the same industrial system. It provides a process-scale perspective on the distribution, transfer, and associated ecological and human health risks of pollutants by investigating phosphate beneficiation and fertilizer production processes in the Mdhilla region simultaneously.

## 2. Materials and Methods

### 2.1. Site Description and Sampling

**Figure 1:**
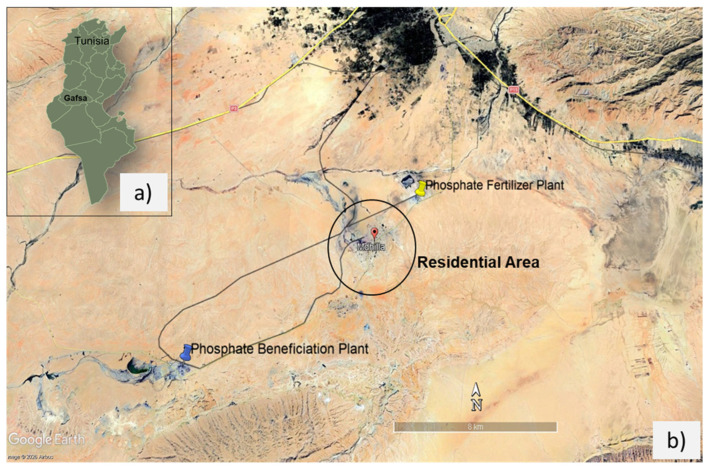
(**a**) Photo showing the location of Gafsa governorate in Tunisia. (**b**) Google Earth Program-generated photo showing the location of the study area in the Gafsa mining basin (southern Tunisia). Base map imagery: Google Earth.

This study was conducted in the Mdhilla region of the Gafsa Governorate in southwestern Tunisia. The area is characterized by an arid to semi-arid climate and occupies a strategic geographic location. It is bordered by the Lala, El Guettar, and Aguila oases to the north, east, and west, respectively. The investigated landscape is visually degraded, with several rivers and wadis disappearing and soils covered by robust saline crusts, and sparse vegetation. These environmental features are strongly associated with the long-term and intensive phosphate industrial activities in the region. Mdhilla represents a unique environmental monitoring hotspot in Tunisia because it is the only region where phosphate beneficiation and chemical fertilizer production co-occur within a limited geographic area (Figure 1). The residential area is situated approximately 3 km from the phosphate fertilizer plant and 10 km from the phosphate beneficiation units. This exposes residents to both industrial solid waste and liquid effluents. This configuration allows a direct comparison of waste from different industrial processes under identical climatic, geological, and socioenvironmental conditions, which is rarely documented in phosphate-producing regions. Sampling campaigns were conducted in September 2024, when ambient temperatures ranged from 28 to 32 °C. The phosphate (PT) stack, generated from phosphate ore beneficiation, and the phosphogypsum (PG) stockpile, which is produced during phosphate fertilizer manufacturing, are located adjacent to the beneficiation and fertilizer plants, respectively. Approximately 1 kg of each solid waste was collected in labeled polyethylene bags (PT and PG) by homogenizing three subsamples. Prior to liquid sampling, polyethylene bottles (5 L capacity) were rinsed three times with the corresponding effluent. Liquid effluents were collected from open channels receiving discharges from the phosphate beneficiation units and the phosphate fertilizer plant with clean plastic beakers. The samples were labeled PBE (phosphate beneficiation effluent) and PFE (phosphate fertilizer effluent), respectively. To prevent metal adsorption and precipitation, effluent samples were collected in acidified bottles for elemental analysis. It should be noted that the current study is based on a single sampling campaign and composite samples collected from representative discharge points. While this approach is capable of capturing the general characteristics of effluents and solid wastes, it does not account for temporal variability associated with operational fluctuations or seasonal changes. All samples were transported to the laboratory in cooled containers and stored at 4 °C until analysis.

### 2.2. Instruments and Reagents

All physicochemical measurements were carried out using calibrated analytical instruments. pH measurements were performed using a digital pH meter (Hanna Instruments, Padovana, Italy). Electrical conductivity (EC), salinity, and total dissolved solids (TDS) were measured with a multiparameter probe (Hach Company, Loveland, CO, USA), while turbidity was determined using a Hach turbidimeter (Hach Company, Loveland, CO, USA). Ion chromatography analyses were conducted using a Metrohm 940 Professional IC Vario system (Metrohm, Herisau, Switzerland) equipped with the appropriate anion and cation exchange columns. Acid digestion of solid samples was performed using a microwave digestion system (Multiwave 3000, Anton Paar, Graz, Austria). Elemental analysis was carried out using a NexION^®^ 5000 multi-quadrupole inductively coupled plasma mass spectrometer (ICP–MS) (PerkinElmer, Waltham, MA, USA), equipped with a PFA nebulizer, a glass cyclone spray chamber, and a triple-cone interface. SEM–EDX analyses were conducted using a JEOL JSM-5310 scanning electron microscope (JEOL Ltd., Tokyo, Japan) coupled with an energy-dispersive X-ray detector. All reagents used were of analytical-reagent grade or higher purity. Hydrochloric acid (HCl, 37%) and nitric acid (HNO_3_, 69%) were used for aqua regia digestion and sample acidification. Ultrapure water (18.2 MΩ·cm), conforming to ASTM Type I specifications (ASTM D1193) [21], was produced using a Milli-Q water purification system (MilliporeSigma, Burlington, MA, USA). Multi-element standard solutions for ICP–MS calibration and rhodium (Rh), which was used as an internal standard, were obtained from certified commercial suppliers. High-purity helium gas (99.999%) was used as the collision gas during the ICP–MS analysis.

### 2.3. Solid Waste Characterization

#### 2.3.1. Determination of Physicochemical Properties

The pH of the phosphate (PT) and phosphogypsum (PG) samples was measured in an aqueous suspension (1:3, *m*/*v*) using a Hanna pH meter after stirring overnight. Organic carbon (C_org_) was determined using the method of Walkley and Black [22]. The percentage of organic matter (OM) was calculated from the organic carbon content. Moisture content was determined gravimetrically by measuring the difference in weight between the fresh sample and the sample that was oven-dried at 105 °C.

#### 2.3.2. Sample Preparation for ICP-MS Analysis

For PT and PG samples, 1.0 g of dried (105 °C) and finely powdered material was placed into clean Teflon digestion vessels and mixed with aqua regia (6 mL HCl, 37% + 2 mL HNO_3_, 69%). Digestions were performed in triplicate using a microwave digestion system (Multiwave 3000, Anton Paar, Graz, Austria) for 1 h. After digestion, the solutions were diluted with ultrapure water to a final volume of 50 mL. The 1:10 dilutions were subsequently prepared, and rhodium (Rh) was added as an internal standard prior to ICP-MS analysis. It should be noted that aqua regia digestion provides pseudo-total metal concentrations, as it may not fully dissolve silicate-bound fractions. Certified reference material (NIST, SRM, 694) was analyzed alongside the samples to ensure analytical accuracy, with recoveries falling within acceptable ranges (85–109%).

#### 2.3.3. Sample Analysis by SEM-EDX

The mineralogical and elemental compositions of solid wastes were examined using a JEOL JSM-5310 scanning electron microscope (SEM) equipped with an energy-dispersive X-ray (EDX) detector. Major elements, including S, F, Fe, Cl, K, Mg, Si, O, P, Na, Ca, and Al, were identified. The samples were oven-dried overnight and cooled in a desiccator. A small fraction of each sample was mounted onto SEM stubs, and surface micrographs were obtained. EDX spectra were recorded from representative areas of each sample, with two measurements per sample. The results are expressed as weight percentages (%).

### 2.4. Effluent Characterization

#### 2.4.1. Water Quality Parameter Measurements

pH was measured using a Hanna pH meter. Electrical conductivity (EC), salinity, and total dissolved solids (TDS) were measured using a Hach multiparameter instrument. Turbidity was determined using a Hach turbidimeter, and total hardness (TH) was calculated using standard methods. All measurements were performed in triplicate.

#### 2.4.2. Determination of Ionic Profiles

The major anions (F^−^, Cl^−^, Br^−^, PO_4_^3−^, SO_4_^2−^, and NO_3_^−^) and cations (Li^+^, Na^+^, K^+^, Mg^2+^, and Ca^2+^) in the effluents were quantified using ion chromatography (IC). The analyses were performed using a Metrohm 940 Professional IC Vario system, in accordance with the protocol described in our previous study [19].

#### 2.4.3. Sample Preparation Before ICP-MS Analysis

Due to differences in turbidity, the effluents were subjected to a process of dilution prior to analysis. A 1:1000 dilution was applied to PBE samples, whereas PFE samples were diluted to 1:100. The dilutions were prepared using an acid matrix consisting of 2% HNO_3_ and 0.5% HCl in order to match the calibration standards. Procedural blanks were prepared using the same acid mixtures.

#### 2.4.4. ICP-MS Analysis

Elemental concentrations were determined using a NexION^®^ 5000 multi-quadrupole ICP-MS (PerkinElmer, Waltham, MA, USA) operating in quantitative mode. Calibration curves were established using multi-element standard solutions in the range of 0 to 300 µg L^−1^. All calibration curves exhibited strong linearity with R^2^ values greater than 0.99. The sample introduction system consisted of a PFA nebulizer coupled to a glass cyclone spray chamber. The ICP-MS was equipped with a triple-cone interface and operated in Kinetic Energy Discrimination (KED) mode using helium gas (99.999%). The instrumental parameters that were employed included an RF power of 1600 W and a plasma gas flow rate of 16 L min^−1^. The limits of detection (LODs) were calculated as the lowest analyte concentrations measured with acceptable statistical certainty.

### 2.5. Quality Assurance and Quality Control (QA/QC)

Quality control procedures were implemented to ensure analytical accuracy and data reliability. These included the analysis of procedural blanks, replicate samples, and spiked samples. The chemicals used in this study were of analytical-reagent grade or higher purity. Glassware and plasticware were soaked in 10% (*v*/*v*) HNO_3_ for at least 24 h, rinsed thoroughly with ultrapure water, and oven-dried prior to use. The analytical reliability of the study was ensured through the implementation of systematic quality control measures. These measures included the regular measurement of blanks and standards, as well as the execution of triplicate analyses for each sample. The recoveries obtained from spiked samples ranged between 92 and 116% for solid waste samples and between 98 and 117% for effluent samples. The slightly elevated recoveries (>100%) may have been attributed to matrix effects or minor spectral interferences associated with the complex composition of phosphate-related samples. The limits of detection for metals in effluents ranged from 0.01 to 0.41 µg L^−1^, while those for solid waste were between 0.0003 and 0.041 mg kg^−1^.

### 2.6. Water Pollution Metrics (WPMs)

#### 2.6.1. Single-Element Pollution Indices

##### Contamination Factor (CF)

The contamination factor (CF) is a metric used to quantify the extent of contamination of a water sample by a specific toxic element. It is computed by calculating the ratio between the measured concentration of the element of interest in the sample (*C_i_*) and the concentration of the same element in the background water (*C_bkg_*). As shown in Formula (1), the background value of potentially toxic elements (PTEs) functions as an indicator, distinguishing between the concentration of naturally occurring PTEs (Table S1) and the anthropogenic influence in a given sample [23]. It should be noted that CF values depend strongly on the selected background reference (borehole water), which may influence the magnitude of calculated contamination indices.

(1) $$\mathrm{CF}=\frac{Ci}{Cbkg}$$

Hakanson [24] categorized the contamination factor into four categories as follows: CF < 1: no contamination; 1 < CF < 2: suspected contamination; 2 < CF < 3.5: slight contamination; 3.5 < CF < 8: moderate contamination; 8 < CF < 27: severe contamination; and CF > 27: extreme contamination.

##### Pollution Index (PI)

The pollution index (PI) quantifies the degree of pollution associated with each individual metal relative to an established permissible concentration.

(2) $$\mathrm{PI}=\frac{Ci}{Si}$$

where *Ci* is the concentration of metal *i* in the effluent (mg/L) and *Si* represents the corresponding standard or permissible guideline value (DPH/APHA). The pollution index is evaluated as follows: PI < 1 indicates low contamination; 1 ≤ PI < 2 indicates moderate contamination; and PI ≥ 2 indicates high pollution [25].

#### 2.6.2. Multi-Element Pollution Indices

##### Contamination Degree (C_deg_)

The level of contamination (C_deg_) was used to assess the total contamination level of the wastewater with various potentially toxic elements (PTEs). C_deg_ is a measure of the total deviation of individual concentrations of PTEs from their corresponding background or reference values. It was calculated using the sum of the contamination factors (Cfs) for individual elements:

(3) $$C_{\deg}\;=\;\sum_{i=1}^{i=n}CF$$

where the contamination factor (*CF*) for each PTE is defined as mentioned above in Formula (1). The degree of contamination was interpreted according to commonly adopted classifications, where increasing C_deg_ values indicate progressively higher levels of contamination and environmental concern. Brady et al. [26] classified the site state based on C_deg_ as follows: C_deg_ < 1.5: unpolluted; 1.5 < C_deg_ < 2: slightly polluted; 2 < C_deg_ < 4: moderately polluted; 4 < C_deg_ < 8: moderately to heavily polluted; 8 < C_deg_ < 16: severely polluted; 16 < C_deg_ < 32: heavily polluted; and C_deg_ > 32: extremely polluted.

##### Potentially Toxic Element Pollution Index (PTEPI)

To complement the contamination degree and provide an integrated assessment of cumulative PTE pollution, the potentially toxic element pollution index (PTEPI) was applied. PTEPI is an adaptation of the Heavy Metal Pollution Index (HPI) originally proposed by Prasad and Bose [27], with the terminology modified to reflect the broader classification of PTEs currently adopted in environmental toxicology. The mathematical formulation and classification criteria of the original HPI were retained without modification. The PTEPI was calculated using the weighted arithmetic approach as follows:

(4) $$\mathrm{PTEPI}\;=\;\frac{\sum Wi\times Qi}{\sum Wi}$$

where *Wi* is the unit weight of the ith PTE and *Qi* is its corresponding sub-index. The unit weight was calculated as the inverse of the permissible concentration:

(5) $$\mathrm{Wi}\;=\;\frac{1}{Si}$$

where *Si* (mg·L^−1^) is the permissible standard concentration for PTE obtained from international water quality guidelines (DPH/APHA). The sub-index (*Qi*) was computed as:

(6) $$\mathrm{Qi}\;=\;\frac{Ci}{Si}\times 100$$

#### 2.6.3. Potential Ecological Risk Index (PERI)

The potential ecological risk index (PERI) was used to quantify the potential ecological threat posed by PTEs, considering both contamination level and metal toxicity [28]. The PERI is determined by multiplying the response toxic factor of a targeted element by its contamination factors, as indicated by the following formula:

(7) ERI = Tr ×CF

(8) $$\mathrm{PERI}=\sum_{i=1}^{n}ERI$$

where CF represents the contamination factor for each contaminant and Tr denotes its toxicity response factor, which is equal to 2, 1, 5, 5, 5, 1, 30, 10, 5 and 10 for Cr, Mn, Co, Ni, Cu, Zn, Cd, Tl, Pb and As, respectively. It is important to note that the potential ecological risk index (PERI) was computed exclusively for elements for which toxicity response coefficients (Tr) are documented in the literature (e.g., Cd, Cr, Ni, Co, Mn, Pb, Zn). Elements such as Al, Fe, V, and Se were excluded from the PERI calculation due to the absence of standardized toxicity coefficients within the Hakanson framework. Therefore, their exclusion can be attributed to methodological concerns rather than being indicative of negligible environmental relevance. The subsequent classification was employed to describe the potential ecological risk index: ERI < 40: low potential ecological risk; 40 ≤ ERI ≤ 80: moderate potential ecological risk; 80 ≤ ERI ≤ 160: considerable potential ecological risk; 160 ≤ ERI ≤ 320: high potential ecological risk; and ERI >320: very high ecological risk at hand for the element in question.

### 2.7. Health Risk Assessment (HRA)

Human health risk assessment (HRA) is a structured approach used to estimate the probability and magnitude of adverse health effects resulting from exposure to environmental contaminants over a defined period [29]. In this study, HRA was applied to evaluate both non-carcinogenic and carcinogenic risks associated with exposure to PTEs present in industrial effluents discharged from phosphate-processing activities. The assessment was conducted in accordance with the United States Environmental Protection Agency (USEPA) risk assessment framework [30], a widely applied methodology for evaluating health risks related to contaminated environmental media.

#### 2.7.1. Exposure Pathways and Receptors

In the investigated area, untreated industrial wastewaters flow continuously through open channels and wadis and spread over surrounding lands, resulting in frequent human contact. Field observations have confirmed that children often engage in swimming or playing in contaminated waters. In contrast, adults—including residents, farmers, and industrial workers—experience incidental or occupational contact during their daily activities. Consequently, two exposure pathways are considered: Firstly, dermal contact is regarded as the primary exposure route, due to direct skin contact with contaminated effluents. Secondly, accidental oral ingestion, considered a secondary exposure route, represents involuntary swallowing of small amounts of contaminated water during recreational or occupational activities. Inhalation exposure is excluded on the basis of the aqueous nature of the contamination and the absence of evidence for aerosolization. Two receptor groups are evaluated: Children constitute the most vulnerable population due to their lower body weight and frequent recreational contact. Adults represent residents and workers experiencing repeated incidental or occupational exposure.

#### 2.7.2. Exposure Assumptions and Frequency

Two exposure pathways were considered: dermal contact and accidental oral ingestion. While dermal contact is likely to occur with greater frequency in open effluent channels, the possibility of accidental ingestion was also taken into consideration due to the potential for hand-to-mouth transfer, particularly in children. This assumption was made in order to reflect realistic contact patterns that were influenced by seasonality, behavioral habits, and occupational activities. Consequently, the exposure frequency (EF) was modified to account for seasonal recreational activities in children and incidental or occupational contact in adults, in accordance with USEPA exposure factor guidance. The inclusion of accidental oral ingestion was deemed necessary to circumvent the underestimation of health risks, despite the fact that effluents are not utilized for drinking-water purposes. The exposure parameters used in the calculations (e.g., exposure frequency, exposure duration, body weight, ingestion rate, and exposed skin area) were selected based on USEPA recommendations and adapted to local conditions. A comprehensive overview of all parameter values and assumptions can be found in Supplementary Table S2. The present health risk assessment does not aim to represent chronic drinking-water exposure; rather, it is intended to evaluate potential human health risks associated with site-specific, intermittent contact with untreated industrial effluents.

#### 2.7.3. Average Daily Dose (ADD) Estimations

The average daily dose (ADD) of PTEs via dermal contact and oral ingestion was calculated using USEPA models. In cases involving dermal exposure, the average daily dose via dermal absorption (ADD_dermal_) was calculated as follows:

(9) $${\mathrm{ADD}}_{\mathrm{dermal}}\;=\;\frac{Cmetal\times SA\times Kp\times ABS\times ET\times EF\times ED\times CF}{BW\times AT}$$

The average daily dose via accidental oral ingestion (ADD_oral_) was calculated as follows:

(10) $${\mathrm{ADD}}_{\mathrm{oral}}\;=\;\frac{CPTE\times Ing\;R\times EF\times ED\times CF}{BW\times AT}$$

where C_PTE_ (mg L^−1^): concentration of the PTE in effluent; SA (cm^2^): exposed skin surface area; Kp (cm h^−1^): dermal permeability coefficient; ABS (unitless): dermal absorption factor; ET (h day^−1^): exposure time; EF (days year^−1^): exposure frequency; ED (years): exposure duration; IR (L day^−1^): ingestion rate; BW (kg): body weight; AT (days): averaging time; and CF: unit conversion factor. Detailed values and sources for all exposure parameters are provided in the Supplementary Table S2.

#### 2.7.4. Non-Carcinogenic Health Risk Assessment

The hazard quotient (HQ) for each PTE and exposure pathway was calculated as:

(11) $${\mathrm{HQ}}_{i}\;=\;\frac{ADD}{RFD}$$

where RfD (mg kg^−1^ day^−1^) is the reference dose specific to each PTE and exposure route (Table S3). The total hazard quotient (THQ) was calculated as the sum of HQ values for all metals and pathways:

(12) $$\mathrm{THQ}\;=\;\sum_{i=1}^{n}{HQ}_{i}$$

AHQ or THQ value < 1 indicates no significant non-carcinogenic health risk, whereas values ≥ 1 suggest potential health concern.

#### 2.7.5. Carcinogenic Health Risk Assessment

The carcinogenic risk (CR) was estimated in order to evaluate the lifetime probability of developing cancer due to exposure to carcinogenic PTEs, following the methodology established by the USEPA. The assessment focused on Cr, As, Cd, and Ni, which are classified as carcinogenic via oral and/or dermal exposure. The carcinogenic risk (CR) for each element and pathway was calculated as follows:

(13) CR = ADD×CSF

where CSF (mg kg^−1^ day^−1^)^−1^ is the cancer slope factor obtained from USEPA-IRIS (Table S4). Chromium was conservatively assumed to be present as Cr(VI). This assumption represents a conservative worst-case scenario. Dermal carcinogenic risk was calculated only for Cr(VI) and inorganic As, as dermal CSFs are unavailable for Cd and Ni. Lead (Pb) was excluded from quantitative carcinogenic risk estimation due to the absence of an official USEPA cancer slope factor and was therefore discussed qualitatively. The total carcinogenic risk (TCR) for each receptor group was calculated as follows:

(14) TCR = ∑CRi

According to USEPA guidelines, CR < 10^−6^ indicates negligible carcinogenic risk, 10^−4^ < CR < 10^−4^ indicates acceptable to tolerable risk, and CR > 10^−4^ indicates unacceptable or high carcinogenic risk requiring regulatory concern or intervention.

## 3. Results

### 3.1. Solid Waste (PT and PG) Characterization

#### 3.1.1. Physicochemical Characterization

The general physicochemical properties of the phosphate beneficiation residue (PT) and phosphogypsum (PG) are summarized in Table 1. Clear contrasts were observed between the two solid byproducts, reflecting their distinct formation processes. PT exhibited a dark gray coloration and a near-neutral pH (7.64 ± 0.01), characteristics of mechanically processed phosphate residues subjected to crushing, grinding, and washing. Conversely, PG exhibited a whitish appearance and a strongly acidic pH of 3.13 ± 0.05, indicative of gypsum precipitation during sulfuric acid digestion in fertilizer production. PT showed higher levels of organic carbon (0.69 ± 0.03%) and organic matter (1.19 ± 0.05%) compared to PG (0.19 ± 0.01% and 0.33 ± 0.02%, respectively), suggesting a significant potential for metallic element retention through organic complexation mechanisms. The moisture content was higher in PG (14.78 ± 0.77%) than in PT (10.6 ± 0.49%), reflecting the hygroscopic nature and water-retention capacity of the gypsum-rich matrix. These physicochemical differences define contrasting geochemical environments that are expected to influence the partitioning of PTEs and mobility in subsequent analyses.

#### 3.1.2. ICP-MS Measurements

The ICP-MS analysis revealed marked differences in the concentrations of PTEs between the phosphate beneficiation residue (PT) and phosphogypsum (PG) (Table 2). In summary, PT exhibited substantially higher concentrations of the most analyzed elements, including V, Cr, Mn, Ni, Cu, Zn, As, Se, Cd, Pb, and Tl, compared to PG. Among these elements, Zn (350 mg/kg), Cr (329 mg/kg), Pb (103 mg/kg), Cd (38.77 mg/kg), and Se (44.08 mg/kg) were found to be particularly enriched in the beneficiation residue. The finding indicates a strong accumulation of PTEs during the mechanical processing of phosphate rock. In contrast, PG exhibited comparatively lower concentrations of these elements, with Zn (72.81 mg/kg), Cr (19.37 mg/kg), Cd (8.93 mg/kg), and Pb (1.89 mg/kg) showing pronounced depletion relative to PT. The consistent reduction in metal concentrations in PG suggested that a significant fraction of trace elements remained associated with beneficiation residues rather than being transferred to the phosphogypsum generated during fertilizer production.

#### 3.1.3. SEM-EDX Profiles

**Figure 2:**
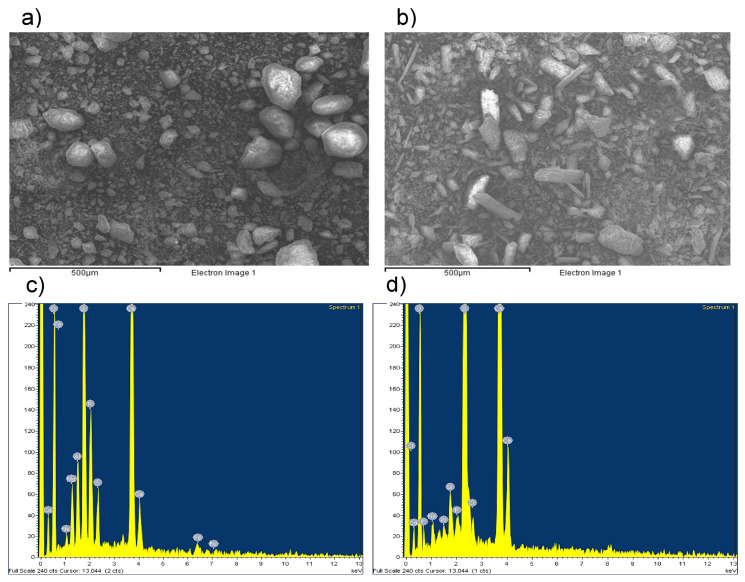
SEM micrographs and corresponding EDX spectra of phosphate industry solid byproducts. (**a**) Phosphate beneficiation residue showing heterogeneous grain sizes and irregular particle morphology resulting from mechanical processing. (**b**) Phosphogypsum characterized by elongated gypsum crystals formed during chemical precipitation in fertilizer production. (**c**,**d**) EDX spectra illustrating the major elemental composition of each matrix, reflecting distinct formation processes that influence heavy metal retention and release behavior.

The contrasting metal concentrations observed between PT and PG are consistent with the morphological and compositional differences identified by SEM–EDX analysis (Figure 2, Tables S5 and S6). SEM images (Figure 2a,b) revealed that PT is composed of heterogeneous, mechanically fragmented particles with irregular surfaces, resulting from crushing, grinding, and washing operations. Such freshly fractured mineral surfaces favor the retention of trace metals initially hosted in phosphate and gangue minerals, including Cd, Pb, Cr, and Zn. These metals are known to substitute for Ca or associate with accessory phases. Conversely, phosphogypsum displays a more homogeneous crystalline morphology, characterized by the predominance of elongated gypsum crystals, as evidenced by SEM observations. Furthermore, EDX spectra (Figure 2c,d) confirmed that the matrix is predominantly composed of Ca, S, and O. This sulfate-dominated structure is less favorable for the retention of numerous trace metals, which may either persist in upstream solid residues or undergo redistribution between the liquid phase and solid matrix during acid digestion. The substantially lower metal concentrations measured in PG by ICP-MS therefore reflect process-dependent partitioning mechanisms rather than simple dilution effects. When considered together, the results of the SEM–EDX and ICP-MS analyses demonstrate that phosphate beneficiation acts as a pivotal stage in concentrating PTEs in solid residues. Concurrently, the process of fertilizer production promotes the partial immobilization of metals within phosphogypsum, thereby reducing their abundance in this byproduct.

### 3.2. Liquid Effluent Characerization

#### 3.2.1. Physicochemical Properties

The physicochemical properties of phosphate beneficiation effluent (PBE) and phosphate fertilizer effluent (PFE), along with their compliance with national (DPH) and international (APHA) discharge standards, are presented in Table 3. Distinct contrasts were observed between the two effluents, reflecting their different industrial origins. PBE exhibited a neutral pH (7.4 ± 0.02), whereas PFE was strongly acidic (2.71 ± 0.02), thus markedly exceeding permissible discharge limits. Electrical conductivity and total dissolved solids exhibited remarkably elevated levels in both effluents, with PFE displaying significantly higher values (52.46 ± 0.03 mS cm^−1^ and 46.04 ± 0.10 g/L, respectively) compared to PBE (9.71 ± 0.04 mS cm^−1^ and 15.16 ± 0.01 g/L), indicating a hypersaline matrix. The two effluents exhibited very high hardness values, particularly PBE (34,107 ± 494 °f), reflecting substantial calcium and magnesium contributions associated with phosphate ore processing. The levels of turbidity in both effluents far exceeded the regulatory thresholds, with PBE exhibiting exceptionally high turbidity (756 ± 7.57 NTU), indicative of abundant suspended solids and fine particulate matter. Overall, the physicochemical characteristics demonstrate that both PBE and PFE are unsuitable for direct discharge into the environment, and provide a chemical basis for the subsequent distribution and mobility of PTEs observed in the effluents.

#### 3.2.2. Ionic Profiling

**Figure 3:**
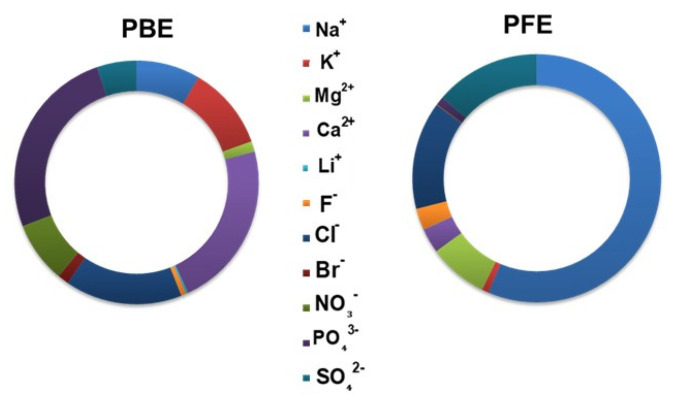
Relative ionic composition (%) of phosphate beneficiation effluent (PBE) and phosphate fertilizer effluent (PFE) determined by ion chromatography (IC). Percentages were calculated from measured ion concentrations to highlight differences in effluent chemical fingerprints.

The ionic profiling of the two effluents was determined by using an ion chromatography analysis. Absolute concentrations are reported in Table 4 and Table S5, while Figure 3 illustrates the relative ionic contributions to highlight compositional differences between effluents. The ionic composition of phosphate beneficiation effluent (PBE) and phosphate fertilizer effluent (PFE) showed pronounced differences, reflecting distinct industrial processes (Table 4). PBE exhibited substantially lower ionic strength, with Ca^2+^ (351.5 mg/L) and Na^+^ (134.9 mg/L) as the dominant cations, and PO_4_^3−^ (406.5 mg/L) and NO_3_^−^ (129.3 mg/L) as the most abundant anions. The concentrations of sulfate and fluoride in PBE were found to be markedly lower than those in PFE, thereby highlighting the absence of sulfuric acid processing during the beneficiation process. In contrast, PFE was characterized by very high concentrations of major dissolved ions, particularly Na^+^ (14,485.6 mg/L), Cl^−^ (3611.5 mg/L), SO_4_^2−^ (3540.5 mg/L), Mg^2+^ (1995 mg/L), Ca^2+^ (823 mg/L), and F^−^ (723 mg/L), indicating a highly saline and sulfate-rich effluent derived from acid digestion of phosphate rock. The relative ionic composition of the two effluents (Figure 3), expressed as the percentage contribution of major ions, highlights distinct chemical fingerprints associated with the beneficiation and fertilizer production processes. The phosphate beneficiation effluent (PBE) exhibits a markedly different ionic profile, characterized by the predominance of Ca^2+^ and PO_4_^3−^, accompanied by moderate contributions from Na^+^, Cl^−^, and NO_3_^−^. Sulfate and fluoride contribute only marginally to the overall ionic balance, reflecting the absence of acid digestion during beneficiation operations. In contrast, the phosphate fertilizer effluent (PFE) is dominated by Na^+^, Cl^−^, and SO_4_^2−^ ions, which together account for the majority of dissolved ions, indicating a highly saline and sulfate-rich matrix derived from sulfuric acid digestion and associated neutralization reactions. Divalent cations, including Mg^2+^ and Ca^2+^, and fluoride represent secondary contributors to the chemical composition of the water sample, while phosphate and nitrate occur only as minor components.

#### 3.2.3. Heavy Metal Composition Determined by ICP/MS Analysis

The concentrations of PTEs in phosphate beneficiation effluent (PBE) and phosphate fertilizer effluent (PFE), as determined by ICP-MS and compared with national (DPH) and international (APHA) discharge standards, are presented in Table 5. Overall, PBE exhibited markedly higher concentrations of most of the elements analyzed in comparison with PFE. Specifically, elevated concentrations were identified for Fe (163.4 ± 0.83 mg/L), Cr (3.09 ± 0.39 mg/L), Cd (0.49 ± 0.004 mg/L), and Pb (0.71 ± 0.082 mg/L). These levels far exceeded the permissible limits for discharge set by regulatory agencies. Despite the strong acidic nature of PFE, dissolved metal concentrations were found to be lower than those measured in PBE. Fe concentrations were found to be close to regulatory limits, while Pb, Cr, and Ni concentrations were comparatively reduced. However, both effluents showed concentrations that exceeded the established guideline values for multiple toxic elements, including Cd, As, Se, and Zn, indicating the possibility of environmental and human health concerns.

### 3.3. PTE Pollution Status of Industrial Effluents

#### 3.3.1. Single Heavy Metal Indices

**Figure 4:**
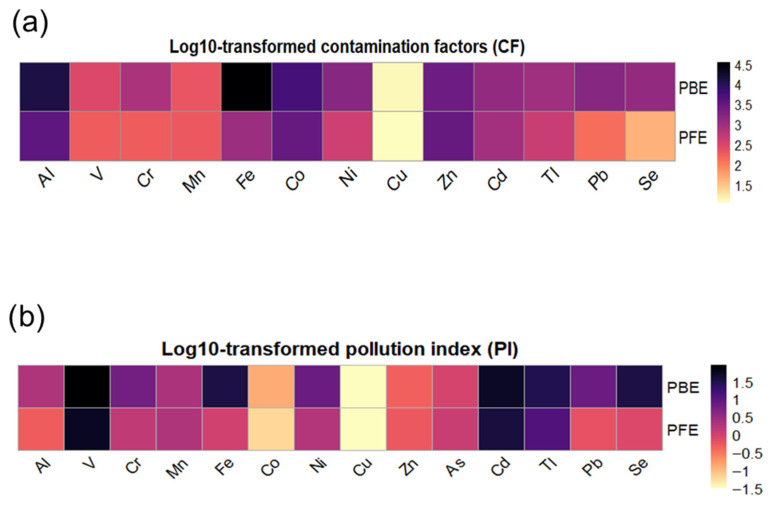
Log_10_-transformed contamination factor (CF) (**a**) and pollution index (PI) (**b**) heatmaps for potentially toxic elements in phosphate beneficiation effluent (PBE) and phosphate fertilizer effluent (PFE).

The contamination factor (CF) and pollution index (PI) heatmaps reveal pronounced metal enrichment in both phosphate beneficiation effluent (PBE) and phosphate fertilizer effluent (PFE), with clear differences in contamination intensity and elemental dominance between effluents (Figure 4, Tables S7 and S8). PBE has been shown to exhibit elevated CF and PI values across a wide range of elements, indicating that there is a stronger contamination signature associated with upstream beneficiation processes. In PBE, Fe and Al show remarkably high CF values (CF > 27), suggesting extreme contamination according to the Hakanson classification. Similarly, Cd, Tl, Zn, Ni, Co, and Pb also demonstrate severe to extreme enrichment. These elements are reflected by elevated PI values, with Cd, Tl, Se, Fe, and V being the primary contributors to pollution relative to regulatory standards (PI ≥ 2). In contrast, Cu exhibits low CF and PI values, suggesting minimal contribution to effluent contamination. The PFE shows a similar but attenuated pattern. Despite the presence of Fe and Al, their CF values are markedly lower than those observed in PBE, indicating either partial dilution or retention during the fertilizer production process. Nevertheless, Cd, Zn, Tl, Pb, and Se continue to exhibit high CF and moderate-to-high PI values, highlighting the persistence of toxic trace elements during downstream processing. Other metals, including Cr, Mn, and V, display moderate contamination levels, while Cu and Co remain within low pollution ranges. The combined CF–PI assessment demonstrates that both effluents are characterized by severe metal contamination, with PBE representing the most critical environmental source. 

#### 3.3.2. Integrated Heavy Metal Indices

**Figure 5:**
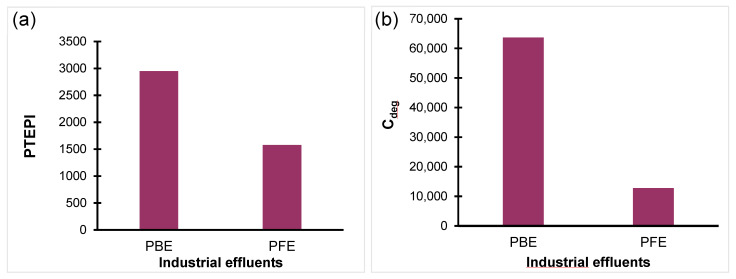
Integrated evaluation of overall metal pollution pressure in phosphate beneficiation and fertilizer effluents. (**a**) Potentially toxic element pollution index (PTEPI); (**b**) contamination degree (C_deg_).

As illustrated in Figure 5, the cumulative contamination degree (C_deg_) and the potential toxic element pollution index (PTEPI) were determined for the industrial effluents under investigation. The C_deg_ values indicate extreme multi-element contamination in both effluents, with markedly higher levels in the phosphate beneficiation effluent (PBE; C_deg_ = 63,660) compared to the phosphate fertilizer effluent (PFE; C_deg_ = 12,815). According to the classification of Brady et al. [26], both values far exceed the threshold for extreme pollution (C_deg_ > 32), confirming severe cumulative metal enrichment, particularly in PBE. In accordance with this pattern, the PTEPI results show a clear contrast between effluents, with PBE exhibiting a substantially higher pollution intensity compared to PFE (Figure 5b). Despite the lower PTEPI values exhibited by PFE, both effluents demonstrated continuously elevated levels, suggesting persistent contamination by potentially toxic elements. The agreement between C_deg_ and PTEPI underscores that phosphate beneficiation constitutes the main source of cumulative toxic metal loading, while downstream fertilizer production mitigates but does not eliminate contamination. C_deg_ is a metric used to quantify cumulative metal enrichment, whereas PTEPI evaluates the resulting pollution severity relative to standards. Together, these metrics offer complementary insight into overall contamination pressure. These findings are fully consistent with those of the CF, PI, and PERI results, providing a robust basis for the subsequent ecological and human health risk assessment.

### 3.4. Ecological Risk Assessment

**Figure 6:**
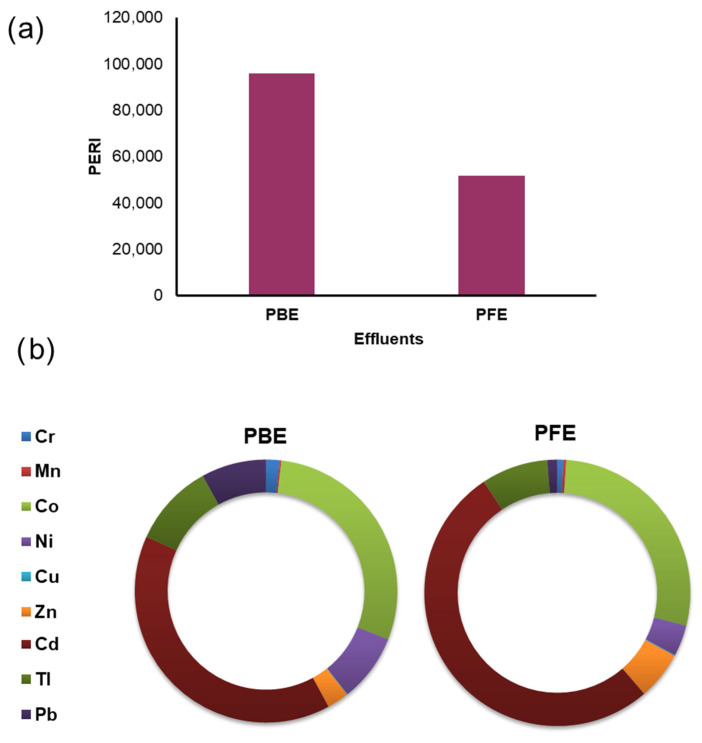
Potential ecological risk associated with potentially toxic elements in industrial effluents: (**a**) total potential ecological risk index (PERI) for phosphate beneficiation effluent (PBE) and phosphate fertilizer effluent (PFE); (**b**) relative contribution of individual potentially toxic elements to PERI in PBE and PFE.

The potential ecological risk index (PERI) revealed extremely high ecological risk levels for both industrial effluents (Figure 6). The phosphate beneficiation effluent (PBE) exhibited a markedly higher PERI value compared to the phosphate fertilizer effluent (PFE), indicating a substantially greater cumulative ecological threat associated with beneficiation activities. In both cases, PERI values far exceeded the threshold for very high ecological risk (PERI > 600), thereby underscoring the severity of metal contamination in the studied effluents (Figure 6a). A comprehensive analysis of the individual metal contributions to PERI revealed that the overall ecological risk was predominantly influenced by a limited number of toxic elements (Figure 6b). In PBE, Cd exhibited the most significant contribution (~40%), followed by Co (~29%), Tl (~10%), Ni, and Pb (~8%). Other metals, including Cr, As, Zn, and Cu, contributed marginally to the total PERI. Similarly, in PFE, Cd remained the predominant contributor (~52%), followed by Co (~28%), with moderate contributions from Zn (~6%), Ni (~4%), and Tl (~8%). The contributions of Cr, As, Cu, and Pb were found to be negligible. The persistence of Cd as the primary contributor in both effluents highlights its critical role in driving ecological risk, despite disparities in overall PERI magnitude between PBE and PFE. Overall, the PERI results demonstrate that although fertilizer effluents exhibit a reduced cumulative ecological risk than beneficiation effluents, both effluent categories pose substantial ecological threats, primarily attributed to a limited group of highly toxic and mobile metals.

### 3.5. Human Risk Assessment (HRA)

**Figure 7:**
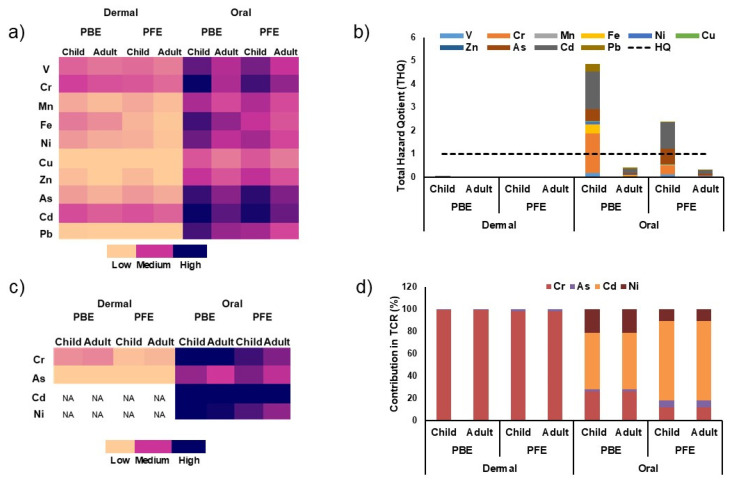
Non-carcinogenic and carcinogenic health risk assessment associated with dermal and oral exposure to metals in phosphate beneficiation effluent (PBE) and phosphate fertilizer effluent (PFE) for children and adults. (**a**) Heatmap of individual hazard quotients (HQs) showing relative non-carcinogenic risk levels for dermal and oral exposure pathways. (**b**) Total hazard quotient (THQ) with stacked contributions of individual metals; the dashed line indicates the USEPA threshold for acceptable non-carcinogenic risk (HQ = 1). (**c**) Heatmap of carcinogenic risk (CR) for carcinogenic metals (Cr, As, Cd, Ni); NA indicates negligible or unavailable dermal carcinogenic risk. (**d**) Relative contributions (%) of individual metals to total carcinogenic risk (TCR) for dermal and oral exposure pathways.

The results of the non-carcinogenic and carcinogenic health risks of the studied industrial effluents are summarized in Figure 7 and Tables S8–S10. The non-carcinogenic risk patterns (Figure 7a,b) demonstrate that oral exposure predominates over dermal exposure for both populations, with children exhibiting consistently higher risk levels compared to adults. For oral intake, the THQ values exceed the safety threshold (HQ = 1) for PBE and, to a lesser extent, for PFE. However, dermal exposure remains well below the threshold. The stacked THQ analysis indicates that a limited number of metals, particularly Cr, Cd, As, and Zn, drive non-carcinogenic risks. The carcinogenic risk results (Figure 7c,d) further highlight the predominance of the oral exposure pathway, while dermal carcinogenic risk is negligible. The total carcinogenic risk is predominantly influenced by Cd and Cr, with secondary contributions from As and Ni, and is consistently higher in children than in adults. Overall, Figure 7 demonstrates that oral exposure to PBE represents the most critical health concern, while PFE shows comparatively reduced but non-negligible non-carcinogenic and carcinogenic risks.

## 4. Discussion

Despite its role as a pivotal economic activity in many countries, including Tunisia, Morocco, Jordan, China, and the United States, high-quality phosphate production and transformation have been linked to significant environmental concerns. These industries have been criticized for their role in discharging various types of waste, including solid waste and liquid effluents [9,19,31,32,33,34,35,36], which have a detrimental impact on water resources and ecosystems. In Tunisia, the Gafsa mining area, particularly the Mdhilla region, has been identified as the most affected region. In this case, in addition to gas emissions, there are solid deposits of phosphate and phosphogypsum, as well as wastewater, released from both the phosphate beneficiation unit and the phosphate fertilizer plant [18,19,34,37]. Similar environmental concerns have been reported in phosphate industrial regions worldwide, including Morocco, China, Jordan, Algeria, West Africa and the United States, where phosphate mining and fertilizer production generate substantial volumes of solid wastes and contaminated effluents [32,38,39,40]. However, the extent of PTE mobilization and environmental impact varies depending on ore composition, processing technologies, and waste management practices. In this context, the Mdhilla industrial complex represents a particularly pertinent case study due to the spatial interconnection of mining, beneficiation and fertilizer production activities within a confined area.

The physichochemical and elemental composition differences between PT and PG are consistent with the mechanical nature of phosphate beneficiation, which concentrates metal-bearing mineral phases within solid residues, as opposed to the chemical transformation processes involved in fertilizer production. Similar observations were reported by El Zrelli et al. [9] for phosphate materials from the Gabes region, where phosphate rock and beneficiation-related materials were found to contain complex mineral assemblages including carbonate fluorapatite, calcite, dolomite, quartz, and trace sulfide phases, such as sphalerite, which acts as a major host for Cd and associated metals. In contrast, phosphogypsum was dominated by synthetic gypsum (CaSO_4_·2H_2_O), with trace metals present as impurities within the sulfate matrix. The agreement between the two studies suggests that the partitioning of metals between PT and PG is primarily governed by mineralogical inheritance and the steps involved in industrial processing rather than being solely influenced by site-specific conditions. However, it is imperative to make a crucial distinction regarding industrial configuration. In fact, the region of Gabes is dedicated mainly to fertilizer production and relies on beneficiated phosphate transported from the Gafsa basin, with no operational phosphate extraction or beneficiation units [9]. Conversely, the Mdhilla region combines phosphate beneficiation and fertilizer production within a limited geographic area [34]. This configuration explains the augmented metal enrichment observed in PT at Mdhilla and highlights the added environmental burden associated with on-site beneficiation activities. The contrasting physicochemical properties of phosphate beneficiation effluent (PBE) and phosphate fertilizer effluent (PFE) provide insight into metal mobilization mechanisms. Despite its near-neutral pH, PBE exhibited markedly higher turbidity and elevated concentrations of most dissolved metals compared to the highly acidic PFE. This finding indicates that acidity alone is not a reliable predictor of metal release in phosphate-processing effluents. The elevated metal concentrations observed in PBE, despite its near-neutral pH, can be attributed to elevated turbidity and the predominance of particle-bound transport. Suspended solids provide extensive reactive surfaces that facilitate metal adsorption and desorption processes, thereby enhancing apparent metal concentrations in bulk water analyses. In contrast, the acidic and sulfate-rich conditions in PFE promote metal co-precipitation and partitioning into solid phases such as phosphogypsum, thereby reducing dissolved metal levels. These observations underscore the significance of phase partitioning and solid–liquid interactions in regulating metal mobility. Recent advances in catalytic and adsorption-based treatment systems further support the role of solid matrices in controlling metal mobility. For instance, the use of Ce–Fe_2_O_3_/Al_2_O_3_ catalysts has been demonstrated to be highly efficient in the removal of Cr(VI) under near-neutral conditions through a combination of adsorption and redox processes, where enhanced electron transfer facilitates the reduction of soluble Cr(VI) to less mobile Cr(III) forms [41]. In a similar manner, Al-based porous materials, including metal–organic framework (MOF) composites, exhibit notable adsorption capabilities attributable to surface complexation and pore–structure interactions, thereby highlighting the importance of solid-phase affinity in pollutant immobilization [42]. These mechanisms are consistent with the observed behavior in phosphate fertilizer effluents, where sulfate-rich and gypsum-dominated matrices promote metal retention through co-precipitation and surface binding, thereby limiting their dissolved concentrations. A clearer linkage between solid residues and wastewater composition can be established by considering the mineralogical and process-related controls governing metal partitioning. Phosphate tailings (PTs), which are characterized by elevated concentrations of carbonate fluorapatite, dolomite, and trace sulfide phases, act as primary sources of PTEs during the beneficiation process. Mechanical processes such as crushing, washing, and flotation enhance the release of particle-bound metals into phosphate beneficiation effluents (PBEs), where elevated turbidity further facilitates metal transport through adsorption–desorption dynamics. Conversely, within the context of fertilizer production, the process of acid digestion of phosphate rock under sulfate-rich conditions leads to the formation of phosphogypsum (CaSO_4_·2H_2_O), which acts as a geochemical sink for multiple metals. This process promotes the co-precipitation, adsorption, and partial structural incorporation of trace elements within the gypsum matrix, thereby reducing their dissolved concentrations in phosphate fertilizer effluents (PFEs). These findings highlight that solid–liquid partitioning of PTEs is primarily controlled by the interplay between mineralogical inheritance and process-induced physicochemical transformations.

The results from the ion chromatography assay revealed that the PBE sample was predominantly composed of calcium and phosphate ions. In contrast, the PFE showed significant enrichment in sulfate and calcium ions, reflecting the occurrence of phosphogypsum formation during the process of acid digestion. The sulfate-rich chemistry of PFE promotes metal co-precipitation and immobilization within sparingly soluble sulfate phases, thereby limiting metal solubilization despite acidic conditions. Conversely, the elevated particulate load and calcium–phosphate-dominated matrix of PBE promote particle-bound metal transport and surface desorption processes, resulting in enhanced metal dispersion in aqueous phases. The higher metal concentrations observed in PBE compared to PFE are consistent with the calcium–phosphate-dominated ionic matrix and elevated turbidity of beneficiation effluents, which favor particle-bound transport and metal desorption, whereas sulfate-rich fertilizer effluents promote metal immobilization through co-precipitation with phosphogypsum. The observed metal concentrations and behavior in Mdhilla effluents differ significantly from those reported for phosphate mine waters in other regions. Al-Hwaiti et al. [32] investigated mine dewatering and flotation waters from the Eshidiya phosphate mine in Jordan and reported detectable levels of several metals, including Cr, Mn, Ni, and Zn. However, these levels were generally below regulatory discharge limits. In this study, the metal occurrence was largely attributed to the natural geochemical interactions between water and phosphate-bearing rocks, with limited industrial processing influence. In contrast, the effluents investigated in the present study originate from active industrial processes involving ore washing, flotation, and acid digestion, which mechanically and chemically enhance metal liberation. As a result, metal concentrations in PBE and, to a lesser extent, PFE were higher than those reported for Jordanian mine waters, underscoring the greater contaminant mobilization potential of industrial effluents compared to passive mine drainage. The occurrence of Tl and Co in the studied materials can be attributed to their association with specific mineral phases present in phosphate deposits. Thallium is typically associated with trace sulfide minerals, including pyrite and sphalerite, and may also occur through substitution in K-bearing phases or association with organic matter. Cobalt is commonly associated with Fe- and Mn-bearing minerals, including iron oxides, carbonates, and sulfide phases, where it can substitute for Fe or Mn within crystal lattices. The results of the present study are in accordance with previous studies performed in the Gafsa phosphatic area and confirmed the high content of HMs in wastewater discharged randomly from numerous phosphate laundries in Metloui [8,16,17]; Moulares, Redeyef and Kef Eddour [15]; and Mdhilla [18]. However, there is a paucity of information regarding the composition of wastewater released from phosphate fertilizer plants in terms of heavy metals. For instance, our earlier study characterized untreated wastewater from a phosphate fertilizer plant in M’dhilla–Gafsa (Tunisia) and reported elevated levels of PTEs, accompanied by evidence of acute biological toxicity, underscoring potential human health risks from direct industrial effluent exposure [19]. 

The immobilization of metals during the production of fertilizer can be explained by several coupled geochemical mechanisms. The wet process of phosphoric acid production generates sulfate-rich conditions that promote the precipitation of phosphogypsum (CaSO_4_·2H_2_O), which acts as a major sink for dissolved metals through co-precipitation during crystal formation. In addition, certain divalent metals such as Cd^2+^ and Pb^2+^ can substitute Ca^2+^ within the gypsum lattice, resulting in their structural incorporation and long-term stabilization. Metals may also be retained through surface adsorption and physical occlusion within growing gypsum crystals. Furthermore, the coexistence of sulfate and residual phosphate species favors the formation of poorly soluble metal-bearing phases (e.g., metal phosphates or hydroxides), which further limits their mobility. These combined processes explain the lower dissolved metal concentrations observed in fertilizer effluents compared to beneficiation wastewater.

Beyond absolute metal concentrations, the application of single and integrated pollution indices provides a more comprehensive assessment of contamination severity and environmental relevance. The contamination factor (CF) and pollution index (PI) have repeatedly indicated that phosphate beneficiation effluent (PBE) is the primary source of metal contamination, exhibiting extreme enrichment of Fe, Al, Cd, Tl, Pb, and Zn. While CF is indicative of the degree of geochemical enrichment relative to background levels, PI places this enrichment in a regulatory context, demonstrating that several toxic elements in PBE exceed permissible discharge limits by orders of magnitude. The concurrence of elevated CF and PI values confirms that metal contamination in PBE is not only geochemically significant but also environmentally hazardous, whereas the lower but still elevated PI values in phosphate fertilizer effluent (PFE) suggest partial attenuation during downstream processing. The integrated indices further emphasize the cumulative nature of contamination. The exceptionally high contamination degree (C_deg_) values calculated for both effluents, particularly PBE, signify extreme multi-element pollution far exceeding commonly adopted classification thresholds [25]. Similarly, the potential toxic element pollution index (PTEPI) reveals a pronounced contrast between effluents, with PBE exhibiting substantially higher pollution intensity than PFE. The agreement between C_deg_ and PTEPI demonstrates that phosphate beneficiation constitutes the principal source of cumulative toxic metal loading, while fertilizer production processes reduce but do not eliminate the overall pollution burden. These integrated indices are particularly relevant in industrial contexts, as they are capable of capturing the combined effects of multiple contaminants, which may act synergistically within the environment. The ecological implications of this cumulative contamination are highlighted by the potential ecological risk index (PERI), which indicates extremely high ecological risk levels for both effluents. The predominance of Cd as the primary contributor to PERI in both PBE and PFE reflects its high toxicity coefficient and strong mobility in aqueous systems, which is consistent with prior findings in phosphate-processing environments [7,8,16]. The contributions from Co, Mn, and, to a lesser extent, Ni and Tl further suggest that ecological risk is governed by a limited group of highly toxic and redox-sensitive elements rather than by overall metal abundance alone. The markedly higher PERI values observed for PBE underscore the critical role of beneficiation processes in amplifying ecological threats. The reduced but still severe PERI associated with PFE indicates persistent risk even after chemical transformation and partial metal immobilization. In this way, many studies emphasize the potential of phosphate-processing activities on different living forms, like soil bacteria communities [43], plants [8,16], and animals [17].

The human health risk assessment reveals marked discrepancies in exposure pathways, population vulnerability, and effluent types. Although dermal contact may represent the most common exposure route in open-channel environments, the risk assessment results clearly demonstrate that accidental oral ingestion is the dominant pathway for both non-carcinogenic and carcinogenic risks. This predominance is attributed to higher ingestion rates and exposure factors, which outweigh dermal absorption contributions. The human health risk assessment revealed clear differences in exposure pathways, population vulnerability, and effluent types. Although dermal contact may represent the most frequent exposure route in open-channel environments, the results indicate that accidental oral ingestion is the dominant pathway for both non-carcinogenic and carcinogenic risks, exceeding dermal contributions by one to two orders of magnitude. This predominance is primarily attributed to higher ingestion rates and associated exposure factors, consistent with previous studies on industrial and mining effluents [32]. Children consistently exhibited higher hazard quotients (HQs), total hazard quotients (THQs), and carcinogenic risks than adults, reflecting lower body weight, greater exposure factors, and increased susceptibility to toxic elements, as widely reported in environmental health assessments [33]. In phosphate beneficiation effluents (PBEs), THQ values for children exceeded the safety threshold (HQ = 1), indicating potential adverse health effects under repeated intermittent exposure, with Cd and Cr identified as the principal contributors due to their high toxicity and cumulative effects. Conversely, phosphate fertilizer effluents (PFEs) exhibited lower THQ values, generally below or near unity, suggesting partial attenuation of health risks during downstream processing. The carcinogenic risk assessment further confirmed these trends, with Cd and Cr as the dominant contributors, followed by As and Ni. Estimated carcinogenic risks for oral exposure to PBE, particularly in children, approached or exceeded the USEPA acceptable range (10^−6^–10^−4^), indicating potential long-term health concerns. In contrast, risks associated with PFE were generally lower and often within acceptable limits, reflecting reduced metal bioavailability due to immobilization within phosphogypsum during fertilizer production. These findings contrast with those reported for phosphate mine waters in Jordan, where HQ and HI values remained below unity [32], highlighting the critical role of industrial processing intensity and site-specific conditions in determining human health risk. The combined evidence from solid waste characterization, effluent chemistry, and ecological and human health risk assessment demonstrates that phosphate beneficiation constitutes a critical stage for PTE release and exposure in the Mdhilla region. The close proximity of residential areas to open effluent channels has been demonstrated to increase the likelihood of repeated human contact, particularly for children engaging in recreational activities. While this study does not provide a comprehensive representation of chronic drinking-water exposure, it underscores the cumulative risks associated with intermittent, site-specific contact with untreated industrial effluents. These findings emphasize the necessity for targeted management strategies that prioritize the beneficiation of effluents, the enhancement of industrial discharge containment, and the augmentation of public awareness concerning the hazards associated with phosphate-processing waste.

Future studies should integrate SEM–EDX characterization and metal speciation analyses to better elucidate solid–liquid partitioning mechanisms. From a management perspective, the elevated metal concentrations observed in phosphate beneficiation effluents (PBEs) highlight the urgent need for targeted treatment strategies. Conventional physicochemical methods, such as neutralization, chemical precipitation, and adsorption [44,45,46,47], can effectively reduce dissolved metal concentrations. In addition, adsorption-based approaches using low-cost materials (e.g., biochar, natural clays, or industrial byproducts) have shown promise for PTE removal from contaminated waters. Nature-based solutions, including constructed wetlands, may also provide sustainable alternatives for long-term treatment under arid conditions. The implementation of such technologies, combined with improved containment of effluent discharge, is essential to mitigate environmental and human health risks in phosphate-processing areas.

Despite the comprehensive assessment provided, this study presents certain limitations that should be acknowledged. The analysis is based on a limited number of sampling campaigns and does not account for potential temporal variations in effluent composition. In addition, the study focuses on total metal concentrations without addressing detailed chemical speciation, which plays a critical role in determining metal mobility and bioavailability. Furthermore, advanced characterization techniques such as SEM–EDX or leaching tests are not performed to elucidate solid-phase metal associations. The assumption that total chromium is present as Cr(VI) represents a conservative worst-case scenario and may overestimate carcinogenic risk in the absence of speciation data. This approach may overestimate carcinogenic risk, and future investigations should incorporate speciation analysis to refine risk assessment accuracy. Although inhalation exposure was not quantitatively assessed, it cannot be excluded under arid conditions where aerosolization of contaminated particles may occur. Future research should integrate these approaches to provide a more refined understanding of PTE transformation mechanisms and environmental behavior.

## 5. Conclusions

This study provides a novel integrated framework by comparatively assessing phosphate beneficiation and fertilizer production within the same industrial setting, enabling a direct evaluation of how each processing stage contributes to PTE release, transformation, and risk. The results demonstrate that phosphate beneficiation constitutes the primary source of metal release, with markedly higher concentrations and pollution indices observed in PBE compared to PFE. The integration of geochemical characterization, pollution indices, ecological risk assessment, and human health risk evaluation highlights the critical role of industrial processing pathways in controlling metal mobility and environmental impact. The findings further indicate that fertilizer production processes contribute to the partial immobilization of metals through their incorporation into phosphogypsum, although residual contamination and associated risks remain significant. The elevated ecological risk (PERI) and human health risk, particularly for children via oral exposure, underscore the urgent need for effective management strategies. Overall, this work emphasizes the necessity of prioritizing wastewater treatment at the beneficiation stage, improving effluent containment, and implementing sustainable remediation strategies to mitigate the environmental and health impacts of phosphate-processing activities.

## Figures and Tables

**Table 1:** General physicochemical characteristics of phosphate beneficiation residue (PT) and phosphogypsum (PG).

Parameters	PT	PG
Color	Dark gray	Whitish
pH	7.64 ± 0.01	3.13 ± 0.05
C_org_ (%)	0.69 ± 0.03	0.19 ± 0.01
MO (%)	1.19 ± 0.05	0.33 ± 0.02
Moisture (%)	10.6 ± 0.49	14.78 ± 0.77

Values are expressed as mean ± standard deviation (*n* = 3). C_org_: organic carbon; OM: organic matter; Moisture (%): gravimetric moisture content.

**Table 2:** Pseudo-total concentrations of PTEs in phosphate beneficiation (PT) residues and phosphogypsum (PG) byproducts in Mdhilla Gafsa, southwestern Tunisia. All concentrations are expressed in mg/kg. Results are presented as the mean of three triplicates and standard deviations.

Elements	PT	PG
V	106 ± 0.75	4.9 ± 0.07
Cr	329 ± 0.28	19.37 ± 0.3
Mn	69.05 ± 0.24	7.19 ± 0.07
Ni	24.16 ± 0.27	3.46 ± 0.03
Cu	9.17 ± 0.09	3.8 ± 0.02
Zn	350 ± 1.17	72.81 ± 0.85
As	9.68 ± 0.1	3.31 ± 0.14
Co	0.19 ± 0.002	2.18 ± 0.07
Se	44.08 ± 2.35	20.07 ± 2.02
Cd	38.77 ± 0.21	8.93 ± 0.13
Pb	103 ± 1	1.89 ± 0.01
Tl	5.36 ± 0.01	2.46 ± 0.001

Metal concentrations are expressed in mg/Kg. Values represent mean ± standard deviation (*n* = 3).

**Table 3:** Physichochemical characterization of effluents discharged by phosphate benefeciation unit (PBE) and phosphate fertilizer industry (PFE) in Mdhilla Gafsa.

Parameters	PBE	PFE	Effluent Discharge Standards	
			DPH	APHA
Color	Greenish gray	Yellow	-	-
Ph	7.4 ± 0.02 ^a^	2.71 ± 0.02 ^b^	6.50–9.50	9.00
EC (ms.cm^−1^)	9.71 ± 0.04 ^a^	52.46 ± 0.03 ^b^	5.00	6.00
TDS (g/L)	15.16 ± 0.01 ^a^	46.04 ± 0.1 ^b^	2.10	-
Hardness (f°)	34,107 ± 494 ^a^	13,238 ± 29.82 ^b^	-	-
Turbidity(NTU)	756 ± 7.57 ^a^	148 ± 1.13 ^b^	70	<30

Values are expressed as mean ± standard deviation (*n* = 3). Different superscript letters (a, b) within the same row indicate statistically significant differences between PBE and PFE at *p* < 0.05.

**Table 4:** Ionic profiling of effluents discharged by phosphate benefeciation unit (PBE) and phosphate fertilizer industry (PFE) in Mdhilla Gafsa.

	PBE	PFE	Effluent Discharge Standards	
			DPH	APHA
Na^+^	134.88 ± 1.25	14,535 ± 61.44	700	-
K^+^	175.76 ± 4.15	233 ± 2.88	50	≤30
Mg^2+^	22.49 ± 0.04	1995 ± 8.48	300	-
Ca^2+^	331 ± 10.6	823 ± 8.13	500	≤200
F^−^	11.04 ± 0.57	723 ± 4.24	-	-
Cl^−^	246.5 ± 4.94	3611 ± 27.57	700	-
Br^−^	23.8 ± 0.98	17.63 ± 0.01	-	-
NO_3_^−^	130.6 ± 1.27	13.34 ± 0.23	-	-
PO_4_^3−^	406.5 ± 3.53	287 ± 12.72	-	-
SO_4_^2−^	83.82 ± 1.23	3540 ± 6.36	600	-
Li^+^	4.48 ± 0.37	10.71 ± 0.02	-	-

Ion concentrations are expressed in mg/L. Values represent mean ± standard deviation (*n* = 3).

**Table 5:** Elemental composition of effluent discharged from phosphate beneficiation (PBE) and effluent discharged from phosphate fertilizers industry (PFE) in Mdhilla Gafsa, southwestern Tunisia, in comparison with national (DPH) and international (APHA) standards. All concentrations are expressed in mg/L. Results are mean of three triplicates and standard deviations.

Elements	PBE	PFE	National Standard (DPH)	International Standard (APHA)
Al	9.03 ± 0.81	2.41 ± 0.18	5.00	-
V	0.89 ± 0.03	0.53 ± 0.01	-	0.01
Cr	3.09 ± 0.39	0.70 ± 0.02	0.5	-
Mn	2.11 ± 0.04	1.83 ± 0.05	1.0	-
Fe	163.4 ± 0.83	5.05 ± 0.06	5.00	≤5.00
Ni	1.5 ± 0.09	0.35 ± 0.09	0.20	-
Cu	0.056 ± 0.005	0.05 ± 0.002	2	≤0.25
Zn	2.19 ± 0.06	2.55 ± 0.03	5.00	≤1.00
As	0.09 ± 0.02	0.11 ± 0.01	0.1	0.01
Co	0.06 ± 0.002	0.03 ± 0.001	0.5	-
Se	1.63 ± 0.47	0.05 ± 0.022	0.05	0.05
Cd	0.49 ± 0.004	0.34 ± 0.007	0.01	≤0.1
Pb	0.71 ± 0.082	0.08 ± 0.005	0.1	≤0.1
Tl	0.05 ± 0.002	0.02 ± 0.001	-	0.002

Metal concentrations are expressed in mg/L. Values represent mean ± standard deviation (*n* = 3).

## Data Availability

The original contributions presented in this study are included in the article/Supplementary Materials. Further inquiries can be directed to the corresponding authors.

## Supplementary Materials

The following supporting information can be downloaded at https://www.mdpi.com/article/10.3390/toxics14050438/s1: Table S1: Background water (borehole) elemental composition by ICP-MS; Table S2: Parameters used in the calculation of the Average Day Dose (ADD); Table S3: Reference doses (RFDs) used in the calculation of the hazard quotients of potentially toxic elements; Table S4: Cancer slope factor (CSF) used in the calculation of the carcinogenic risk of potentially toxic elements; Table S5: EDX results of elements present in phosphate beneficiation effluent (PBE). Results are presented as weight percentages; Table S6: EDX results of elements present in phosphate fertilizer effluent (PFE). Results are presented as weight percentages; Table S7: Contamination factors of potentially toxic elements in both PBE and PFE; Table S8: Pollution indices of potentially toxic elements in both PBE and PFE; Table S9: ADD calculations of PTEs (V, Cr, Mn, Fe, Ni, Cu, Zn, As, Cd, and Pb) in PBE and PFE; Table S10: Hazard quotients of elements (V, Cr, Mn, Fe, Ni, Cu, Zn, As, Cd, and Pb) in PBE and PFE; Table S11: Carcinogenic risks of elements (Cr, Ni, As and Cd) in PBE and PFE.
