# Supplementary material for: Potentially Toxic Elements in Phosphate Processing: A Comparative Assessment of Solid Wastes and Effluents from Beneficiation and Fertilizer Production in Southwest Tunisia
Source: Toxics. 2026 May 15;14(5):438. doi: 10.3390/toxics14050438 (PMC13211432; doi:10.3390/toxics14050438)
Supplement: Supplementary file 1 [file toxics-14-00438-s001.zip › toxics-4228549-supplementary.pdf]

Supplementary materials

Table S1: Background water (Borehole) elemental composition by ICP-MS. All concentrations are expressed in mg/L.

Element	Concentration
Al	0.0007±0
V	0.0031±0.0003
Cr	0.0041±0.0015
Mn	0.0098±0.0002
Fe	0.0047±0.0004
Co	0±0
Ni	0.0013±0.0009
Cu	0.0045±0.0001
Zn	0.0009±0.0003
As	0±0
Se	0.0013±0.0004
Cd	0.0004±0.0001
Tl	0.0001±0
Pb	0.0005±0.0001

Table S2: Parameters used in the calculation of the Average Day Dose (ADD)

Parameter	Symbol	Children	Adults	Unit	Description	Source
Exposure frequency	EF	120	180	Days.year^{-1}	Intermittent exposure reflecting seasonal recreational (children) and incidental/occupational (adults) contact	This study (USEPA Exposure Factors Handbook, **2011**)
Exposure time	ET	1.0	0.5	h.day^{-1}	Average daily duration of skin contact	This study
Exposure duration	ED	6	24	years	Exposure period for children and adults	USEPA Risk Assessment Guidance, **1989**
Body weight	BW	15	70	kg	Average body weight	USEPA Exposure Factors Handbook, **2011**
Averaging time (non-carcinogenic)	AT_nc	2190	8760	days	ED × 365	USEPA Risk Assessment Guidance, **1989**
Averaging time (carcinogenic)	ATc	25,550	25,550	days	Lifetime averaging time (70 years)	USEPA Risk Assessment Guidance, **1989**
Ingestion rate	IR	0.10	0.05	L.day^{-1}	Accidental ingestion of contaminated water	USEPA Exposure Factors Handbook, **2011**
Exposed skin area	SA	2800	5100	cm^2	Partial-body dermal contact (lower limbs, hands, partial arms)	This study (USEPA Dermal Exposure Assessment, **2004**)
Dermal permeability coefficient	Kp	Metal-specific	Metal-specific	cm.h^{-1}	Skin permeability coefficient	USEPA Dermal Exposure Assessment, **2004**
Dermal absorption factor	ABS	Metal-specific	Metal-specific	-	Fraction absorbed through skin	USEPA Dermal Exposure Assessment, **2004**
Unit conversion factor	CF	0.001	0.001	L.cm^{-3}	Unit conversion factor	USEPA Risk Assessment Guidance, **1989**

Table S3: References doses (RFD) used in the calculation of the hazard quotients of potentially toxic elements

Metal	RfD_{oral}	ABS_GI	RfD_dermal	Source
Cd	0.001	0.025	Derived	USEPA IRIS
Cr(VI)	0.003	0.02	Derived	USEPA IRIS
Ni	0.02	0.04	Derived	USEPA IRIS
As	0.0003	0.95	Derived	USEPA IRIS
Cu	0.04	0.4	Derived	USEPA IRIS
Zn	0.3	0.2	Derived	USEPA IRIS
Pb	–	–	–	Not available

Table S4: Cancer Slope Factor (CSF) used in the calculation of the carcinogenic risk of potentially toxic elements

Metal	Exposure route	CSF	Source
Cr(VI)	Oral	0.5	USEPA IRIS
Cr(VI)	Dermal	20	USEPA IRIS
As	Oral	1.5	USEPA IRIS
As	Dermal	3.66	USEPA IRIS
Cd	Oral	6.1	USEPA IRIS
Ni	Oral	0.84	USEPA IRIS
Pb	–	–	Not available

Table S5: EDX results of elements present in phosphate beneficiation effluent (PBE). Results are presented as weight percentages

Element	App Conc.	Intensity Corrn.	Weight%	Weight% Sigma	Atomic %
O K	11.36	0.5369	57.55	1.59	74.12
Na K	0.44	0.6403	1.88	0.43	1.68
Mg K	0.19	0.6154	0.82	0.31	0.70
Al K	0.41	0.7348	1.51	0.26	1.15
Si K	1.49	0.8294	4.90	0.39	3.59
P K	0.50	1.1840	1.15	0.29	0.77
S K	3.72	0.8966	11.28	0.61	7.25
Cl K	0.33	0.7263	1.23	0.25	0.71
K K	0.25	1.0217	0.67	0.20	0.35
Ca K	6.45	0.9599	18.26	0.81	9.39
Fe K	0.23	0.8101	0.76	0.36	0.28
Totals			100.00		

Table S6: EDX results of elements present in phosphate fertilizer effluent (PFE). Results are presented as weight percentages

Element	App Conc.	Intensity Corrn.	Weight %	Weight % Sigma	Atomic%
O K	12.18	0.4559	49.61	1.75	66.48
F K	0.40	0.1364	5.48	1.61	6.19
Na K	0.32	0.6257	0.94	0.29	0.88
Al K	0.11	0.7450	0.27	0.14	0.21
Si K	0.35	0.8579	0.75	0.17	0.57
P K	0.25	1.2910	0.35	0.19	0.24
S K	10.02	0.9628	19.32	0.78	12.92
Cl K	0.25	0.7112	0.65	0.19	0.39
Ca K	11.70	0.9594	22.64	0.89	12.11
Totals			100.00		

Table S7: Contamination factors of potentially toxic elements in both effluent PBE and PFE

	**PBE**	**PFE**
CF Al	12803	3419
CF V	294	175
CF Cr	793	183
CF Mn	215	187
CF Fe	34738	1075
CF Co	55192	2878
CF Ni	1604	387
CF Cu	12.93	11.19
CF Zn	2574	3014
CF Cd	1269	893
CF Tl	981	418
CF Pb	1537	126
CF Se	1238	43.81
C_{deg}	**63 659**	**12 815**

Table S8: Pollution indices of potentially toxic elements in both effluent PBE and PFE

	**PBE**	**PFE**
PI Al	1.81	0.48
PI V	89.59	53.47
PI Cr	6.19	1 .41
PI Mn	2.11	1.84
PI Fe	32.69	1.01
PI Co	0.12	0.06
PI Ni	7.53	1.79
PI Cu	0.03	0.03
PI Zn	0.44	0.51
PI Cd	49.33	34.66
PI Tl	27.52	11.88
PI Pb	7.18	0.61
PI Se	32.72	0.77

Table S9: ADD calculations of PTEs (V, Cr, Mn, Fe, Ni, Cu, Zn, As, Cd and Pb) in effluents PBE and PFE.

	**PBE**				**PFE**			
	**Children**		**Adult**		**Children**		**Adult**	
	**Dermal**	**Oral**	**Dermal**	**Oral**	**Dermal**	**Oral**	**Dermal**	**Oral**
**V**	5.49789E-08	1.47E-03	1.79874E-08	1.75E-04	3.28127E-08	8.79E-04	1.07353E-08	1.05E-04
**Cr**	3.7973E-07	5.09E-03	1.2423E-07	6.05E-04	8.6718E-08	1.16E-03	2.8371E-08	1.38E-04
**Mn**	1.2957E-07	3.47E-03	4.2391E-08	4.13E-04	1.1271E-07	3.02E-03	3.6876E-08	3.59E-04
**Fe**	1.0031E-05	2.69E-01	3.2817E-06	3.20E-02	3.1036E-07	8.31E-03	1.0154E-07	9.90E-04
**Ni**	1.8495E-08	2.48E-03	6.051E-09	5.88E-04	4.3932E-09	2.95E-04	1.4373E-09	7.00E-05
**Cu**	3.417E-09	9.1528E-05	1.1179E-09	1.0896E-05	3.0817E-09	8.2544E-05	1.0082E-09	9.8267E-06
**Zn**	8.0943E-08	3.61E-03	2.6482E-08	4.30E-04	9.3962E-08	4.19E-03	3.0742E-08	4.99E-04
**As**	5.5659E-09	1.49E-04	1.821E-09	1.77E-05	7.2108E-09	1.93E-04	2.3592E-09	2.30E-05
**Cd**	2.30E-05	8.11E-04	9.904E-09	9.65E-05	2.1271E-08	5.70E-04	6.9592E-09	6.78E-05
**Pb**	4.4034E-09	1.18E-03	1.4407E-09	1.40E-04	3.7489E-10	1.00E-04	1.2265E-10	1.20E-05

Table S10: Hazard quotients of elements (V, Cr, Mn, Fe, Ni, Cu, Zn, As, Cd and Pb) in effluents PBE and PFE.

	**PBE**				**PFE**			
	**Children**		**Adult**		**Children**		**Adult**	
	**Dermal**	**Oral**	**Dermal**	**Oral**	**Dermal**	**Oral**	**Dermal**	**Oral**
**HQ V**	6.11E-04	1.64E-01	2.00E-04	1.95E-02	3.65E-04	9.77E-02	1.19E-04	1.16E-02
**HQ Cr**	5.06E-03	**1.70E+00**	1.66E-03	2.02E-01	1.16E-03	3.87E-01	3.78E-04	4.61E-02
**HQ Mn**	9.2549E-06	2.48E-02	3.0279E-06	2.95E-03	8.051E-06	2.16E-02	2.634E-06	2.57E-03
**HQ Fe**	1.43E-04	3.84E-01	4.69E-05	4.57E-02	4.43E-06	1.19E-02	1.45E-06	1.41E-03
**HQ Ni**	2.3119E-05	1.24E-01	7.5638E-06	1.47E-02	5.4915E-06	2.94E-02	1.7967E-06	3.50E-03
**HQ Cu**	8.5426E-07	1.31E-03	2.7949E-07	1.56E-04	7.7041E-07	1.18E-03	2.5206E-07	1.40E-04
**HQ Zn**	2.6981E-06	1.20E-02	8.8273E-07	1.43E-03	3.1321E-06	1.40E-02	1.0247E-06	1.66E-03
**HQ As**	1.8553E-05	4.97E-01	6.07E-06	5.92E-02	2.4036E-05	6.44E-01	7.8639E-06	7.66E-02
**HQ Cd**	2.42E-03	**1.62E+00**	7.92E-04	1.93E-01	1.70E-03	**1.14E+00**	5.57E-04	1.36E-01
**HQ Pb**	1.2581E-06	3.37E-01	4.1162E-07	4.01E-02	1.0711E-07	2.87E-02	3.5044E-08	3.42E-03
**THQ**	8.29E-03	**4.86E+00**	2.71E-03	4.00E-01	3.27E-03	**2.37E+00**	1.07E-03	2.83E-01

Table S11: Carcinogenic risks of elements (Cr, Ni, As and Cd) in effluents PBE and PFE

	PBE				PFE			
	Children		Adult		Children		Adult	
	**Dermal**	**Oral**	**Dermal**	**Oral**	**Dermal**	**Oral**	**Dermal**	**Oral**
**Cr**	6,5096E-07	2,18E-04	8,52E-07	1,04E-04	1,4866E-07	4,9774E-05	1,9455E-07	2,3702E-05
**Ni**	-	1,78E-04	-	8,4927E-05	-	4,2363E-05	-	2,0173E-05
**As**	1,7461E-09	1,9168E-05	2,2851E-09	9,1278E-06	2,2621E-09	2,4833E-05	2,9604E-09	1,1825E-05
**Cd**	-	4,24E-04	-	2,02E-04	-	2,98E-04	-	1,42E-04
**TCR**	6,52704E-07	8,39E-04	8,54E-07	4,00E-04	1,51E-07	4,15E-04	1,98E-07	1,98E-04
